# Association of Framingham Steatosis Index with Albuminuria: A cross-sectional study

**DOI:** 10.1371/journal.pone.0337104

**Published:** 2025-11-20

**Authors:** Dongli Huang, Huan Zou, You Zhang

**Affiliations:** 1 Dongli Huang, Bishan hospital of Chongqing medical university (Bishan Hospital of Chongqing), Bishan District, Chongqing, China; 2 You Zhang, Chongqing Nanchuan District People’s Hospital, Nanchuan District, Chongqing, China; National University of Sciences and Technology, PAKISTAN

## Abstract

**Background:**

The Framingham Steatosis Index (FSI) is a diagnostic indicator of hepatic steatosis. Although prior studies have established associations between hepatic steatosis and chronic kidney disease (CKD) and between FSI and CKD, the association between FSI and proteinuria remains unexplored. This study investigated the association between FSI and albuminuria, addressing this research gap.

**Patients and methods:**

Data were obtained from the National Health and Nutrition Examination Survey (NHANES) database. The association between FSI and albuminuria was examined using multivariable logistic regression and stratified analyses. Nonlinearity was assessed using smoothing curves, and inflection points were located with a recursive algorithm. Subgroup analyses were conducted to evaluate the consistency of the association between FSI and albuminuria across different strata. Finally, propensity score matching (PSM) was applied to reduce potential confounding and enhance the robustness of the findings.

**Results:**

In model 3, which adjusted for all covariates, the odds ratio (OR) for the association between FSI and albuminuria was 1.13 (95% CI: 1.09–1.18). Smooth curve fitting demonstrated a U-shaped relationship between FSI and albuminuria. Threshold analysis identified an inflection point at an FSI value of −3.22 to further characterize this relationship. Subgroup analyses showed directionally consistent associations across strata. The U-shaped relationship between FSI and albuminuria remained robust after applying PSM.

**Conclusion:**

Our study identified a U-shaped relationship between FSI and albuminuria.

## Introduction

Albuminuria, typically defined as an elevated urinary albumin-to-creatinine ratio (ACR), is a sensitive early indicator of kidney injury. As an early marker of kidney injury, albuminuria often precedes a decline in glomerular filtration rate and indicates the onset of glomerular endothelial dysfunction [1]. Clinically, even mild elevations in urinary albumin are associated with accelerated progression of chronic kidney disease (CKD) and increased risk of adverse cardiovascular outcomes [2,3]. In public health, albuminuria is widely used for screening and surveillance, as it identifies subclinical kidney disease and correlates with increased morbidity and mortality [4,5]. Given its prognostic and epidemiological significance, albuminuria is a key marker for assessing early-stage kidney disease at the population level.

The Framingham Steatosis Index (FSI) is a noninvasive scoring system that estimates hepatic fat content using routinely collected clinical and biochemical parameters, including age, sex, body mass index (BMI), triglyceride levels, presence of hypertension and diabetes, and the alanine aminotransferase (ALT) to aspartate aminotransferase (AST) ratio [6]. Since its development, the FSI has demonstrated reliability as a surrogate marker of hepatic steatosis [6,7]. It has attracted growing interest for its potential applications beyond liver disease, particularly in metabolic and cardiovascular risk assessment [8,9]. Concurrently, the link between fatty liver and kidney injury has garnered increasing research interest. This association may reflect shared pathophysiological mechanisms involving the liver and the kidney, such as inflammation and endothelial dysfunction. Meta-analyses have demonstrated that non-alcoholic fatty liver disease (NAFLD) significantly increases the risk of CKD [10], independent of traditional risk factors. Moreover, an association between FSI and CKD has also been reported [11].

Despite accumulating evidence linking hepatic steatosis and FSI to CKD, no study has directly examined the association between FSI and albuminuria. Clarifying this association is essential to determine whether a simple, noninvasive hepatic steatosis score can be a marker for early kidney injury in the general population. The National Health and Nutrition Examination Survey (NHANES) provides a large, nationally representative, and well-characterized dataset. Using NHANES data, we investigated the association between FSI and albuminuria to provide new epidemiological evidence supporting this line of inquiry.

## Methods

### Data and study participants

**Fig 1:**
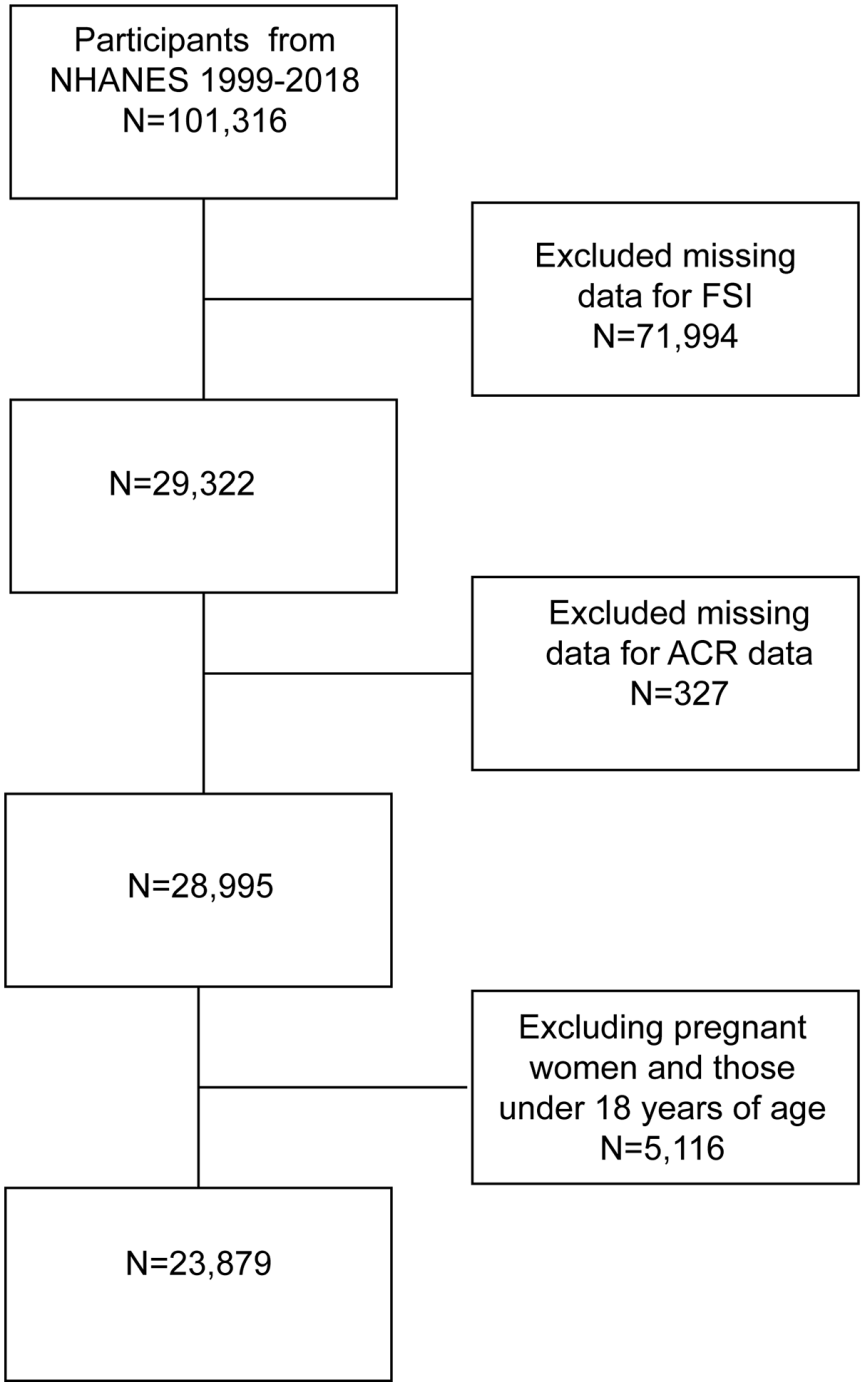
Flowchart of sample selection 1999-2018.

The NHANES is a population-based program that collects health and nutrition data from a representative sample of the U.S. civilian population. Data were collected with a multistage probability sampling design that incorporated structured household interviews, mobile examination center assessments, and laboratory testing. This study utilized de-identified data from NHANES, a publicly available database maintained by the National Center for Health Statistics (NCHS), part of the Centers for Disease Control and Prevention (CDC). The NHANES data collection protocol received approval from the NCHS Research Ethics Review Board (ERB). As this study involved secondary analysis of anonymized, publicly available data, further Institutional Review Board (IRB) approval was not required. NCHS obtained informed consent from all participants during initial data collection; no additional consent was necessary for this analysis. 101,316 participants were initially identified from the 1999–2018 NHANES dataset. We excluded 71,994 participants with missing data on variables required to compute the FSI, including sex, age, hypertension status, diabetes status, triglycerides (TG), AST, ALT, and BMI. Additionally, 327 participants missing ACR data were excluded. Finally, 5,116 pregnant women and participants younger than 18 years were excluded, resulting in a final analytic sample of 23,879 participants, as illustrated in Figure 1. All data used in this study were publicly available (https://www.cdc.gov/nchs/nhanes/).

### Definition of FSI

The FSI developed by Long et al. in 2016 was used in our analysis. The formula is as follows:

FSI = −7.981 + 0.011 × age (years) − 0.146 × sex (female = 1, male = 0) + 0.173 × BMI (kg/m²) + 0.007 × TG (mg/dL) + 0.593 × hypertension (yes = 1, no = 0) + 0.789 × diabetes (yes = 1, no = 0) + 1.1 × ALT:AST ratio ≥ 1.33 (yes = 1, no = 0).

AST, ALT, and TG levels were assessed by trained specialists following standardized protocols established by the NCHS and guided by CDC procedures. Lipid levels were measured from peripheral blood samples collected in the morning after an overnight fast of at least 8 hours. Serum triglyceride and AST levels were measured enzymatically. Serum or plasma ALT levels were determined using kinetic rate assays.

### Outcome ascertainment

The primary outcome of this study was albuminuria, defined as an ACR > 30 mg/g[12]. ACR was calculated by dividing urinary albumin (mg) by urinary creatinine (g). Blood and urine samples were collected from NHANES participants at standardized mobile examination centers. Urinary albumin and creatinine levels were measured in spot urine samples using a solid-phase fluorescence immunoassay and a modified Jaffe kinetic method. These measurements were obtained directly from the NHANES dataset, which utilized automated biochemical analyzers including the Roche Cobas 6000 and Roche Modular P Chemistry Analyzer.

### Assessment of covariates

Covariates included age, sex, race/ethnicity, educational attainment, marital status, poverty-to-income ratio (PIR), BMI, physical activity level, smoking status, alcohol use, diabetes, serum albumin (ALB), estimated glomerular filtration rate (eGFR), uric acid (UA), hypertension, and hyperlipidemia. Physical activity was classified as vigorous or moderate, each coded as yes or no. Current smokers were defined as participants who had smoked at least 100 cigarettes in their lifetime and continued smoking at the time of the survey. Former smokers were those who had smoked at least 100 cigarettes in their lifetime but had quit before the study. Participants who had smoked fewer than 100 cigarettes in their lifetime were considered non-smokers. Non-drinkers were defined as participants who had consumed fewer than 12 alcoholic beverages in their lifetime or any given year. Former drinkers were those who had consumed ≥12 alcoholic drinks in their lifetime or any year but had not consumed alcohol in the past 12 months. Current drinkers were those who had consumed ≥12 alcoholic beverages in their lifetime or any one year and at least one alcoholic drink in the past 12 months. Diabetes was defined as self-reported physician diagnosis, fasting blood glucose ≥7.0 mmol/L, glycosylated hemoglobin (HbA1c) ≥6.5%, or a 2-hour plasma glucose ≥11.1 mmol/L after a 75-g oral glucose tolerance test. Hypertension was defined as self-reported diagnosis, use of antihypertensive medication, systolic blood pressure ≥130 mmHg, or diastolic blood pressure ≥80 mmHg. Hyperlipidemia, as determined by the National Cholesterol Education Program, included TG ≥ 150 mg/dL, total cholesterol ≥200 mg/dL, low-density lipoprotein cholesterol (LDL-C) ≥130 mg/dL, or high-density lipoprotein cholesterol (HDL-C) ≤50 mg/dL (≤40 mg/dL for men).

### Statistical analysis

Analyses were conducted with full consideration of NHANES’s multistage probability design. The design strata (SDMVSTRA), primary sampling units (SDMVPSU), and the fasting subsample weights were specified. Because the key biomarkers were obtained in the morning fasting subsample, WTSAF4YR was used for 1999–2002 and WTSAF2YR for 2003–2018. When pooling ten 2-year cycles (1999–2018), multi-cycle weights were constructed as (2/10) ×WTSAF4YR for 1999–2002 and (1/10) ×WTSAF2YR for each subsequent 2-year cycle. In the baseline characteristics table, continuous variables were presented as means with standard deviation (SD), and categorical variables as proportions. Differences between albuminuria and non-albuminuria groups were assessed using the Kruskal–Wallis test for continuous variables and the chi-square test for categorical variables. Multivariable logistic regression was applied to evaluate the association between FSI and the prevalence of albuminuria. Participants were divided into three FSI tertiles (T1–T3). Three models were constructed: Model 1 (unadjusted); Model 2 (adjusted for age, sex, and ethnicity); and Model 3 (further adjusted for BMI, education, marital status, PIR, albumin, UA, hyperlipidemia, diabetes, alcohol consumption, hypertension, vigorous activity, moderate activity, smoking, and eGFR). Smoothed curve fitting and threshold analysis were used to assess the nonlinearity between FSI and albuminuria. A segmented (two-piecewise) linear regression model was fitted when nonlinear relationships were observed to evaluate threshold effects across intervals. Subgroup analyses were performed to examine the consistency of the association between FSI and albuminuria across strata. Finally, propensity score matching (PSM) was conducted using the nearest-neighbor method to match albuminuria and non-albuminuria participants in a 1:1 ratio. Covariates from Model 3 were used to estimate the propensity score for matching. In addition, sensitivity analyses were performed by further adjusting for hepatitis status and medication use (lipid-lowering drugs, antihypertensives, and antidiabetics), as well as by re-estimating the associations after excluding covariates overlapping with components of the FSI formula. All statistical analyses were performed using EmpowerStats (X&Y Solutions, Inc., http://www.empowerstats.com) and R software (The R Foundation, http://www.R-project.org). A two-sided P value < 0.05 was considered statistically significant.

### Language editing

The manuscript was edited for readability, style, and grammatical accuracy using Grammarly, an artificial intelligence–based tool for non-generative copyediting.

## Results

### Association between FSI and albuminuria

As presented in Table 1, the study included 23,879 participants aged ≥18 years, among whom 2,975 (12.46%) had albuminuria. Participants with albuminuria had higher prevalence of hypertension, diabetes, and reduced eGFR (<60 mL/min/1.73 m²) compared to those without albuminuria (P < 0.05). Albuminuria participants also exhibited higher FSI values than their non-albuminuria counterparts. Detailed baseline characteristics for both groups are summarized in Table 1.

**Table 1:** Baseline characteristics of participants.

Characteristics	Non-albuminuria	albuminuria	P-value
N	20904	2975	
UA, mg/ml	5.44 ± 1.38	5.86 ± 1.68	<0.001
ALB, g/L	42.54 ± 3.31	41.31 ± 3.79	<0.001
ALT, U/L	25.40 ± 27.76	24.49 ± 19.19	<0.001
AST	25.26 ± 22.49	26.19 ± 18.38	0.130
FSI	−1.37 ± 1.69	−0.64 ± 1.98	<0.001
Gender, n (%)			0.065
Male	10496 (50.21%)	1440 (48.40%)	
Female	10408 (49.79%)	1535 (51.60%)	
Age, n (%)			<0.001
<60	14865 (71.11%)	1297 (43.60%)	
>=60	6039 (28.89%)	1678 (56.40%)	
Race, n (%)			<0.001
Mexican American	3804 (18.20%)	613 (20.61%)	
Other Hispanic	1777 (8.50%)	229 (7.70%)	
Non-Hispanic White	9189 (43.96%)	1191 (40.03%)	
Non-Hispanic Black	4220 (20.19%)	695 (23.36%)	
Other Race	1914 (9.16%)	247 (8.30%)	
Education, n (%)			<0.001
Under high school	4961 (23.73%)	1076 (36.17%)	
High school or equivalent	4425 (21.17%)	674 (22.66%)	
College graduate or above	11518 (55.10%)	1225 (41.18%)	
Marital Status, n (%)			<0.001
Married or living with partner	12797 (61.22%)	1646 (55.33%)	
Living alone	8107 (38.78%)	1329 (44.67%)	
PIR			<0.001
<1.3	5845 (27.96%)	995 (33.45%)	
>=1.3, < 3.5	9008 (43.09%)	1410 (47.39%)	
>=3.5	6051 (28.95%)	570 (19.16%)	
eGFR, n (%)			<0.001
<60	1161 (5.55%)	688 (23.13%)	
>=60	19743 (94.45%)	2287 (76.87%)	
Smoke, n (%)			<0.001
Current smokers	4112 (19.67%)	576 (19.36%)	
Nonsmokers	12035 (57.57%)	1522 (51.16%)	
Former smokers	4757 (22.76%)	877 (29.48%)	
Diabetes, n (%)			<0.001
No	17882 (85.54%)	1666 (56.00%)	
Yes	3022 (14.46%)	1309 (44.00%)	
Vigorous activity, n (%)			<0.001
No	13166 (62.98%)	2334 (78.45%)	
Yes	7738 (37.02%)	641 (21.55%)	
Moderate activity, n (%)			<0.001
No	11857 (56.72%)	1941 (65.24%)	
Yes	9047 (43.28%)	1034 (34.76%)	
Drink, n (%)			<0.001
Current drinkers	15082 (72.15%)	1757 (59.06%)	
Nondrinkers	2531 (12.11%)	456 (15.33%)	
Former drinkers	3291 (15.74%)	762 (25.61%)	
Hypertension, n (%)			<0.001
No	10727 (51.32%)	739 (24.84%)	
Yes	10177 (48.68%)	2236 (75.16%)	
Hyperlipidemia, n (%)			<0.001
No	8774 (41.97%)	1053 (35.39%)	
Yes	12130 (58.03%)	1922 (64.61%)	
BMI, n (%)			<0.001
<30	13837 (66.19%)	1702 (57.21%)	
>=30	7067 (33.81%)	1273 (42.79%)	

UA uric acid, FSI framingham steatosis index, PIR poverty-to-income ratio, BMI body mass index, eGFR estimating glomerular filtration rate.

### Association between FSI and albuminuria

As shown in Table 2, higher FSI levels were associated with increased odds of albuminuria in Model 1 (OR = 1.24; 95% CI: 1.21–1.26). This association remained statistically significant after adjustment in Model 2 (OR = 1.19; 95% CI: 1.16–1.21) and Model 3 (OR = 1.13; 95% CI: 1.09–1.18). Participants in the highest FSI tertile had 10% higher odds of albuminuria than those in the lowest tertile (OR = 1.10; 95% CI: 0.92–1.30); however, the dose–response trend across tertiles was not statistically significant (P for trend = 0.108).

**Table 2:** Association of FSI with albuminuria in the population.

	Model 1 OR (95% CI)	Model 2 OR (95% CI)	Model 3 OR (95% CI)
**Albuminuria**	1.24 (1.21, 1.26)	1.19 (1.16, 1.21)	1.13 (1.09, 1.18)
**FSI tertiles**			
T1	Ref	Ref	Ref
T2	1.80 (1.62,2.00)	1.11 (0.99, 1.25)	0.90 (0.79, 1.03)
T3	2.74 (2.47, 3.03)	1.77 (1.59, 1.97)	1.10 (0.92, 1.30)
**P for trend**	<0.001	<0.001	0.108

OR: odds ratio; 95% CI: 95% confidence interval; Model 1: No covariates were adjusted; Model 2: Adjusted for age, gender, and race; Model 3: Adjusted for age, gender, race, BMI, education, marital status, PIR, albumin, uric acid, hyperlipidemia, diabetes, alcohol consumption, hypertension, vigorous activity, moderate activity, smoking, and eGFR.

**Fig 2:**
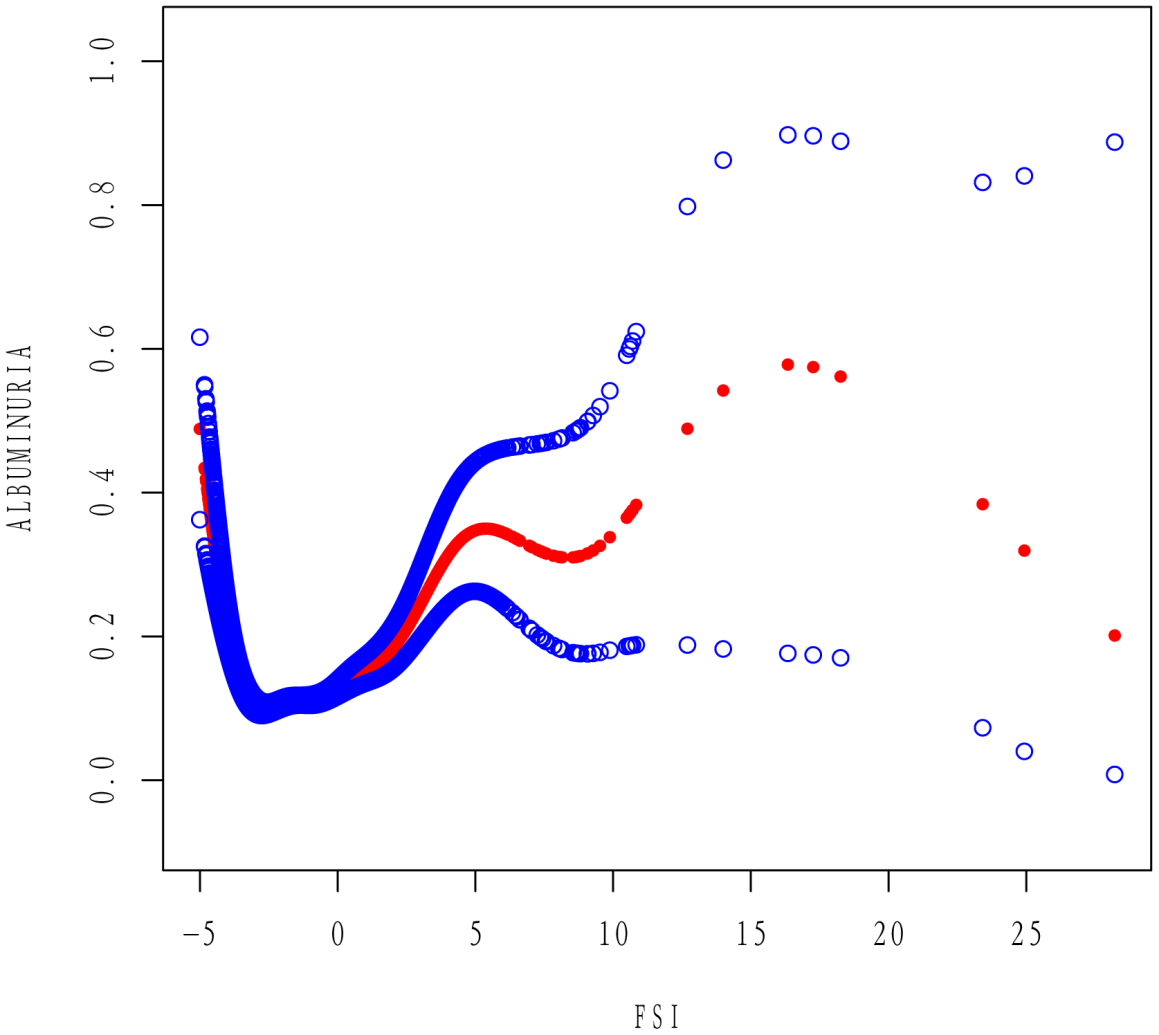
Smooth curve fitting of FSI and albuminuria.

After full adjustment in Model 3 and application of smoothed curve fitting, a U-shaped association between FSI and the prevalence of albuminuria was observed (Fig 2). Threshold effect analysis (Table 3) revealed that when FSI was below −3.22, each 1-unit increase in FSI was associated with a 74% decrease in the odds of albuminuria (OR = 0.26; 95% CI: 0.19–0.34; P < 0.001), whereas when FSI exceeded −3.22, each 1-unit increase in FSI was associated with a 15% increase in the odds of albuminuria (OR = 1.15; 95% CI: 1.10–1.20; P < 0.001).

**Table 3:** Threshold effects of FSI on albuminuria analyzed using linear regression models.

	Adjusted OR (95% CI), P-value
**FSI vs Albuminuria**	
Fitting by the standard linear model	1.13 (1.09, 1.18) <0.001
Fitting by the two-piecewise linear model	
FSI	
Inflection point	−3.22
FSI ＜ −3.22	0.26 (0.19, 0.34) <0.001
FSI ＞ −3.22	1.15 (1.10, 1.20) <0.001
Log likelihood ratio	<0.001

### Subgroup analysis

Subgroup analyses were conducted to explore potential interactions between FSI and albuminuria prevalence across demographic and clinical strata, including sex, age, BMI, PIR, hypertension, diabetes, hyperlipidemia, physical activity, education, marital status, smoking, and alcohol consumption. The association between FSI and albuminuria remained directionally consistent across all subgroups (Table 4).

**Table 4:** Subgroup analysis for the association between FSI and albuminuria.

Character	OR (95% CI)	*P*-value	P for interaction
**BMI**			0.004
<30	1.04 (0.99, 1.10)	0.119	
>= 30	1.15 (1.10, 1.20)	<0.001	
**PIR**			0.076
Below 1.3	1.09 (1.05, 1.15)	<0.001	
1.3-3.5	1.09 (1.05, 1.14)	0.000	
Over 3.5	1.16 (1.10, 1.23)	<0.001	
**Drink**			0.204
Current drinkers	1.12 (1.08, 1.16)	<0.001	
Nondrinkers	1.06 (0.99, 1.13)	0.101	
Former drinkers	1.10 (1.04,1.16)	<0.001	
**Hypertension**			0.001
Yes	1.05 (1.00, 1.10)	0.074	
No	1.14 (1.10, 1.18)	<0.001	
**Diabetes**			0.123
Yes	1.08 (1.04, 1.13)	<0.001	
No	1.13 (1.08, 1.18)	<0.001	
**Vigorous activity**			0.892
Yes	1.10 (1.06, 1.15)	<0.001	
No	1.11 (1.06, 1.16)	<0.001	
**Moderate activity**			0.081
Yes	1.09 (1.05,1.13)	<0.001	
No	1.13 (1.09,1.19)	<0.001	
**Gender**			0.002
Male	1.17 (1.12, 1.22)	<0.001	
Female	1.05 (0.99, 1.10)	0.094	
**Smoke**			0.448
Current smokers	1.13 (1.07, 1.18)	<0.001	
Nonsmokers	1.09 (1.05, 1.14)	<0.001	
Former smokers	1.12 (1.06,1.18)	<0.001	
**Age**			0.274
<60	1.04 (0.92, 1.17)	0.523	
>=60	1.11 (1.07, 1.15)	<0.001	
**Marital status**			0.152
Married or living with partner	1.12 (1.08, 1.17)	<0.001	
Living alone	1.08 (1.04, 1.13)	<0.001	
**Hyperlipidemia**			<0.001
Yes	1.02 (0.96, 1.08)	0.524	
No	1.13 (1.09, 1.17)	<0.001	

OR: odds ratio; 95% CI: 95% confidence interval; Age, gender, race, BMI, education, marital status, PIR, albumin, UA, hyperlipidemia, diabetes, alcohol consumption, hypertension, vigorous activity, moderate activity, smoking, and eGFR were adjusted.

### PSM analysis

After PSM, 5,762 participants were included, comprising 2,881 individuals in both the albuminuria and non-albuminuria groups. Descriptive analyses of the matched population indicated no significant differences between groups for most covariates, except for race and eGFR (S1 Table). Multivariable logistic regression was conducted on the matched sample, and the results are presented in S2 Table. Across all three models, FSI remained significantly associated with increased odds of albuminuria (Model 1: OR = 1.05; 95% CI: 1.02–1.08; Model 2: OR = 1.05; 95% CI: 1.02–1.08; Model 3: OR = 1.14; 95% CI: 1.07–1.21). Smoothed curve fitting and threshold effect analysis conducted in the matched population (S3 Table and S1 Fig) confirmed that the U-shaped relationship between FSI and albuminuria persisted.

### Sensitivity analyses

In sensitivity analyses, the U-shaped association between FSI and albuminuria remained significant after further adjustment for hepatitis status and medication use (S4 Table and S2 Fig). A similar pattern was observed after excluding covariates that overlapped with components of the FSI formula (S5 Table and S3 Fig).

## Discussion

This extensive NHANES-based cross-sectional study identified a statistically significant association between the FSI and albuminuria [12]. In fully adjusted models, higher FSI values were associated with increased odds of albuminuria (adjusted OR = 1.13; 95% CI: 1.09–1.18). Notably, dose–response analysis revealed a U-shaped relationship, with an inflection point at FSI = −3.22, indicating increased prevalence of albuminuria at both low and high FSI levels. This nonlinear association remained consistent after propensity score matching to control for potential confounders. These findings suggest a complex relationship between FSI and early renal injury.

Our findings are broadly consistent with previous evidence linking NAFLD to kidney injury. For example, in the Multi-Ethnic Study of Atherosclerosis (MESA), greater liver fat accumulation on computed tomography (CT) was associated with an elevated risk of albuminuria. Among individuals without diabetes or hypertension, a 10-unit decrease in liver CT attenuation, indicating increased hepatic fat, was linked to higher prevalence and incidence of albuminuria [13]. Similarly, a population-based study in China using the fatty liver index (FLI) observed a stepwise increase in albuminuria across FLI quartiles. Participants in the highest quartile had a significantly greater risk of elevated albumin excretion than those in the lowest quartile (OR = 2.30; 95% CI: 1.36–3.90) [14]. In both diabetic and non-diabetic populations, NAFLD has been associated with an increased risk of albuminuria. For instance, among Korean patients with type 2 diabetes, those with ultrasound-detected hepatic steatosis had a substantially higher prevalence of albuminuria (32.1% vs. 6.8%). Moreover, steatosis was independently associated with albuminuria (adjusted OR = 1.88) [15]. Even among non-diabetic men, NAFLD (defined as FLI ≥ 60) was associated with approximately twice the odds of low-grade albuminuria (OR = 2.3) [16]. Accordingly, our findings of a positive association between FSI and albuminuria in a nationally representative US population complement and extend prior research.

The pattern of our findings—particularly the U-shaped association—echoes those from recent analyses examining FSI in relation to renal outcomes. In an extensive NHANES-based study, Jiang et al. reported a curvilinear relationship between FSI and CKD, with a similar inflection point (FSI = −3.21) [11]. Below this threshold, FSI was inversely associated with CKD risk (OR = 0.25), whereas a positive association was observed (OR = 1.19). The near-identical inflection point (FSI = −3.22) suggests a potentially reproducible threshold in the relationship between steatosis and albuminuria. This U-shaped pattern may partly explain the inconsistent findings in the literature regarding NAFLD and kidney disease. For instance, a recent U.S. study reported that MAFLD alone was not significantly associated with albuminuria, whereas elevated risk (OR = 1.73) was observed only in those with evidence of hepatic fibrosis [17]. In that study, advanced liver disease—rather than simple steatosis—was the primary driver of renal risk; this aligns with our finding that the positive association intensifies at higher FSI values.

The U-shaped association between FSI and albuminuria suggests the existence of two distinct risk phenotypes at the lower and upper extremes of the FSI spectrum. FSI is calculated using age, sex, BMI, triglyceride levels, hypertension, diabetes status, and the ALT to aspartate aminotransferase AST ratio. Accordingly, low FSI values (e.g., below −3.22) are typically observed in lean (low BMI), non-hypertensive, non-diabetic, and often older or female. This phenotype does not necessarily reflect good health. Instead, it may represent an “atypical low metabolic” state, such as sarcopenia, malnutrition, or frailty in older adults. Sarcopenia and malnutrition have been independently associated with an elevated risk of albuminuria [18]. Glomerular barrier integrity may be compromised in such frail individuals, and microvascular regulation may be impaired. For instance, impaired blood pressure regulation, such as orthostatic hypotension, is associated with a 1.7-fold increased risk of albuminuria [19], and chronic reductions in renal perfusion or autoregulatory capacity may contribute to glomerular injury [20]. Thus, individuals with low FSI—often frail or malnourished—may exhibit impaired nephron integrity, hypotension, and diminished capillary support [21], all of which may contribute to albuminuria even in the absence of traditional metabolic disorders [22].

In contrast, higher FSI values (i.e., > −3.22) reflect the presence of multiple metabolic risk factors. These include elevated BMI (overweight or obesity), high triglyceride levels, hypertension, diabetes, and increased ALT/AST ratios, which reflect hepatic steatosis and insulin resistance. Collectively, these features characterize metabolic syndrome, a condition well recognized to impair renal function [23]. Obesity and insulin resistance contribute to glomerular hyperfiltration and intraglomerular hypertension, whereas chronic hypertension further elevates glomerular pressure and imposes mechanical strain on the basement membrane [24]. Dyslipidemia (e.g., hypertriglyceridemia) and hepatic steatosis promote systemic inflammation and endothelial dysfunction, whereas insulin resistance exacerbates oxidative stress. Insulin resistance, particularly, is associated with chronic low-grade inflammation and contributes to hypertension, endothelial injury, and dyslipidemia [25]. These pathophysiological processes compromise glomerular barrier function, leading to increased permeability. In summary, at higher FSI levels, the cumulative burden of obesity, dyslipidemia, hypertension, and diabetes, together with the pro-inflammatory state reflected by elevated ALT/AST ratios [26], synergistically contributes to glomerular injury and increased albuminuria.

Taken together, these considerations may help explain the U-shaped pattern: very low FSI identifies a low-BMI/frail subgroup prone to perfusion-related glomerular vulnerability, whereas very high FSI reflects classic metabolic syndrome with its own injurious effects on the kidney. Both extremes, via different pathways, can ultimately increase albuminuria risk.

Our study has several strengths, including a large, nationally representative NHANES sample and multiple robustness checks (multivariable adjustment and propensity score matching). Nonetheless, important limitations merit emphasis. First, the cross-sectional design precludes causal inference; the observed association between FSI and albuminuria may reflect reverse causation or shared determinants rather than a directional effect. Second, albuminuria was assessed from a single spot urine sample and can be influenced by short-term factors (e.g., recent exercise, fever/infection, hydration), so persistence could not be confirmed. Third, despite extensive adjustment, residual confounding is likely, particularly from unmeasured or imprecisely measured lifestyle and inflammatory factors (e.g., dietary patterns and low-grade systemic inflammation). Finally, because some covariates considered for adjustment may overlap with components used to construct FSI, partial overadjustment and collinearity are possible; such bias would generally attenuate the estimated associations. These caveats should be considered when interpreting our findings.

Our findings suggest that FSI, calculated from routine clinical variables, might help identify individuals at elevated risk of kidney injury. Albuminuria is a recognized early marker of renal and cardiovascular disease, so FSI is a readily available score that may aid risk stratification and early identification. Since FSI is computed from standard metrics (age, BMI, TG, ALT, AST, diabetes, and hypertension status), it could be integrated into clinical practice without additional testing. In the future, prospective studies should examine whether baseline FSI predicts incident albuminuria. Moreover, mechanistic research is needed to clarify how steatosis‐related factors contribute to glomerular damage. For example, whether interventions that control FSI (through weight loss, lipid control, or glycemic management) also reduce albuminuria risk would be a valuable question.

## Conclusions

Our study highlights the complex relationship between FSI and albuminuria in the general US population. Nonlinear analyses further revealed that this relationship is U-shaped, revealing that controlling FSI to a specific range may help reduce the risk of early kidney damage and provide valuable insights for future interventions.

## Supporting information

S1 Table Baseline characteristics of participants after PSM. (DOCX)

S2 Table Association of FSI with albuminuria after PSM. (DOCX)

S3 Table Threshold effects of FIS on albuminuria analyzed using linear regression models after PSM. (DOCX)

S4 Table Association of FSI with albuminuria after additional adjustment for hepatitis and medication use (lipid-lowering drugs, antihypertensives, and antidiabetics). (DOCX)

S5 Table Association of FSI with albuminuria after excluding overlapping components of FSI from covariate adjustment. (DOCX)

S1 Fig U-shaped relationship between FSI and Albuminuria after PSM. (TIF)

S2 Fig U-shaped relationship between FSI and albuminuria after additional adjustment for hepatitis and medication use. (TIF)

S3 Fig U-shaped relationship between FSI and albuminuria after excluding overlapping components of FSI from covariate adjustment. (TIF)
