# Supplementary material for: Association of Framingham Steatosis Index with Albuminuria: A cross-sectional study
Source: PLoS One. 2025 Nov 20;20(11):e0337104. doi: 10.1371/journal.pone.0337104 (PMC12633878; doi:10.1371/journal.pone.0337104)
Supplement: S1 Table — (DOCX) [file pone.0337104.s001.docx]

S1 Table: Baseline characteristics of participants after PSM.

| characteristics | No-albuminuria | albuminuria | P-value |
| --- | --- | --- | --- |
| N | 2881 | 2881 |  |
| UA, (mg/ml) | 5.79 ± 1.52 | 5.80 ± 1.64 | 0.838 |
| ALB, (g/L) | 41.63 ± 3.41 | 41.47 ± 3.64 | 0.314 |
| FSI | -0.83 ± 1.64 | -0.66 ± 1.98 | 0.018 |
| Gender, n(%) |  |  | 0.510 |
| Male | 1375 (47.73%) | 1400 (48.59%) |  |
| Female | 1506 (52.27%) | 1481 (51.41%) |  |
| Age, n(%) |  |  | 0.354 |
| <60 | 1321 (45.85%) | 1286 (44.64%) |  |
| >=60 | 1560 (54.15%) | 1595 (55.36%) |  |
| Race, n(%) |  |  | <0.001 |
| Mexican American | 494 (17.15%) | 591 (20.51%) |  |
| Other Hispanic | 284 (9.86%) | 219 (7.60%) |  |
| Non-Hispanic White | 1285 (44.60%) | 1159 (40.23%) |  |
| Non-Hispanic Black | 594 (20.62%) | 672 (23.33%) |  |
| Other Race | 224 (7.78%) | 240 (8.33%) |  |
| Education, n(%) |  |  | 0.307 |
| Under high school | 957 (33.22%) | 1009 (35.02%) |  |
| High school or equivalent | 693 (24.05%) | 659 (22.87%) |  |
| College graduate or above | 1231 (42.73%) | 1213 (42.10%) |  |
| Marital Status，n(%) |  |  | 0.633 |
| Married or living with partner | 1628 (56.51%) | 1610 (55.88%) |  |
| living alone | 1253 (43.49%) | 1271 (44.12%) |  |
| PIR |  |  | 0.108 |
| <1.3 | 938 (32.56%) | 943 (32.73%) |  |
| >=1.3, <3.5 | 1317 (45.71%) | 1374 (47.69%) |  |
| >=3.5 | 626 (21.73%) | 564 (19.58%) |  |
| eGFR,n(%) |  |  | <0.001 |
| <60 | 461 (16.00%) | 607 (21.07%) |  |
| >=60 | 2420 (84.00%) | 2274 (78.93%) |  |
| Smoke，n(%) |  |  | 0.505 |
| Current smokers | 549 (19.06%) | 559 (19.40%) |  |
| Nonsmokers | 1527 (53.00%) | 1484 (51.51%) |  |
| Former smokers | 805 (27.94%) | 838 (29.09%) |  |
| Diabetes，n(%) |  |  | 0.336 |
| No | 1702 (59.08%) | 1666 (57.83%) |  |
| Yes | 1179 (40.92%) | 1215 (42.17%) |  |
| Vigorous activity,n(%) |  |  | 0.468 |
| No | 2222 (77.13%) | 2245 (77.92%) |  |
| Yes | 659 (22.87%) | 636 (22.08%) |  |
| Moderate activity, (n%) |  |  | 0.720 |
| No | 1861 (64.60%) | 1874 (65.05%) |  |
| Yes | 1020 (35.40%) | 1007 (34.95%) |  |
| Drink，n(%) |  |  | 0.719 |
| Current drinkers | 1739 (60.36%) | 1729 (60.01%) |  |
| Nondrinkers | 445 (15.45%) | 431 (14.96%) |  |
| Former drinkers | 697 (24.19%) | 721 (25.03%) |  |
| Hypertension,n(%) |  |  | 0.471 |
| No | 763 (26.48%) | 739 (25.65%) |  |
| Yes | 2118 (73.52%) | 2142 (74.35%) |  |
| Hyperlipidmia, n(%) |  |  | 0.721 |
| 0 | 1041 (36.13%) | 1028 (35.68%) |  |
| 1 | 1840 (63.87%) | 1853 (64.32%) |  |
| BMI, n(%) |  |  | 0.134 |
| <30 | 1713 (59.46%) | 1657 (57.51%) |  |
| >=30 | 1168 (40.54%) | 1224 (42.49%) |  |
