# Supplementary material for: Association of Framingham Steatosis Index with Albuminuria: A cross-sectional study
Source: PLoS One. 2025 Nov 20;20(11):e0337104. doi: 10.1371/journal.pone.0337104 (PMC12633878; doi:10.1371/journal.pone.0337104)
Supplement: S2 Table — (DOCX) [file pone.0337104.s002.docx]

S2 Table: Association of FSI with albuminuria after PSM

|  | **Model 1**  **OR 95% CI** |  | **Model 2**  **OR 95% CI** | **Model 3**  **OR 95% CI** |
| --- | --- | --- | --- | --- |
| albuminuria | 1.05 (1.02, 1.08) |  | 1.05 (1.02, 1.08) | 1.14 (1.07, 1.21) |

OR: odds ratio

95% CI: 95% confidence interval

Model 1: No covariates were adjusted

Model 2: Adjusted for age, gender, and race

Model 3: Adjusted for age, gender, race, body mass index, education, marital status, PIR, albumin, uric acid, hyperlipidemia, diabetes, alcohol consumption, hypertension, vigorous activity, moderate activity, smoking, and eGFR.

.

|  |  |  |  |  |  |
| --- | --- | --- | --- | --- | --- |
