# Supplementary material for: Association of Framingham Steatosis Index with Albuminuria: A cross-sectional study
Source: PLoS One. 2025 Nov 20;20(11):e0337104. doi: 10.1371/journal.pone.0337104 (PMC12633878; doi:10.1371/journal.pone.0337104)
Supplement: S3 Table — (DOCX) [file pone.0337104.s003.docx]

S3 Table: Threshold effects of FIS on albuminuria analyzed using linear regression models after PSM.

|  | **Adjusted OR (95% CI), P Value** |
| --- | --- |
| **FSI vs Albuminuria**  Fitting by the standard linear model | 1.14 (1.07, 1.21) <0.0001 |
| Fitting by the two-piecewise linear model |  |
| FSI |  |
| Inflection point | -3.35 |
| FSI＜-3.35 | 0.19 (0.11, 0.32) <0.0001 |
| FSI＞-3.35  Log likelihood ratio | 1.15 (1.08, 1.23) <0.0001  <0.001 |
