# Supplementary material for: Association of Framingham Steatosis Index with Albuminuria: A cross-sectional study
Source: PLoS One. 2025 Nov 20;20(11):e0337104. doi: 10.1371/journal.pone.0337104 (PMC12633878; doi:10.1371/journal.pone.0337104)
Supplement: S4 Table — (DOCX) [file pone.0337104.s004.docx]

S4 Table: Association of FSI with albuminuria after additional adjustment for hepatitis and medication use (lipid-lowering drugs, antihypertensives, and antidiabetics)

|  | **Model 1**  **OR 95% CI** |  | **Model 2**  **OR 95% CI** | **Model 3**  **OR 95% CI** |
| --- | --- | --- | --- | --- |
| albuminuria | 1.24 (1.21, 1.27) |  | 1.19 (1.16, 1.21) | 1.13 (1.08, 1.17) |
| T1 | Ref |  | Ref | Ref |
| T2 | 1.82 (1.63, 2.03) |  | 1.13 (1.00, 1.26) | 0.90 (0.79, 1.03) |
| T3 | 2.77 (2.50, 3.06) |  | 1.78 (1.60, 1.99) | 1.06 (0.89, 1.27) |
| P for trend | <0.0001 |  | <0.0001 | 0.3007 |

OR: odds ratio

95% CI: 95% confidence interval

Model 1: No covariates were adjusted

Model 2: Adjusted for age, gender, and race

Model 3: Adjusted for age, gender, race, body mass index, education, marital status, PIR, albumin, uric acid, hyperlipidemia, diabetes, alcohol consumption, hypertension, vigorous activity, moderate activity, smoking, eGFR, hepatitis, lipid-lowering drugs, antihypertensives, and antidiabetics.
