# Supplementary material for: Association of Framingham Steatosis Index with Albuminuria: A cross-sectional study
Source: PLoS One. 2025 Nov 20;20(11):e0337104. doi: 10.1371/journal.pone.0337104 (PMC12633878; doi:10.1371/journal.pone.0337104)
Supplement: S5 Table — (DOCX) [file pone.0337104.s005.docx]

S5 Table: Association of FSI with albuminuria after excluding overlapping components of FSI from covariate adjustment.

|  | **Model 1**  **OR 95% CI** | **Model 2**  **OR 95% CI** |
| --- | --- | --- |
| Albuminuria | 1.24 (1.21, 1.26) | 1.14 (1.12, 1.17) |
| T1 | Ref | Ref |
| T2 | 1.80 (1.62, 2.00) | 1.14 (1.01, 1.27) |
| T3 | 2.74 (2.47, 3.03) | 1.59 (1.42, 1.79) |
| P for trend | <0.0001 | <0.0001 |

OR: odds ratio

95% CI: 95% confidence interval

Model 1: No covariates were adjusted.

Model 2: Adjusted for race, education, marital status, PIR, albumin, uric acid, alcohol consumption, vigorous activity, moderate activity, smoking and eGFR.
