# Supplementary figures and images for: Association of Framingham Steatosis Index with Albuminuria: A cross-sectional study
Source: PLoS One. 2025 Nov 20;20(11):e0337104. doi: 10.1371/journal.pone.0337104 (PMC12633878; doi:10.1371/journal.pone.0337104)

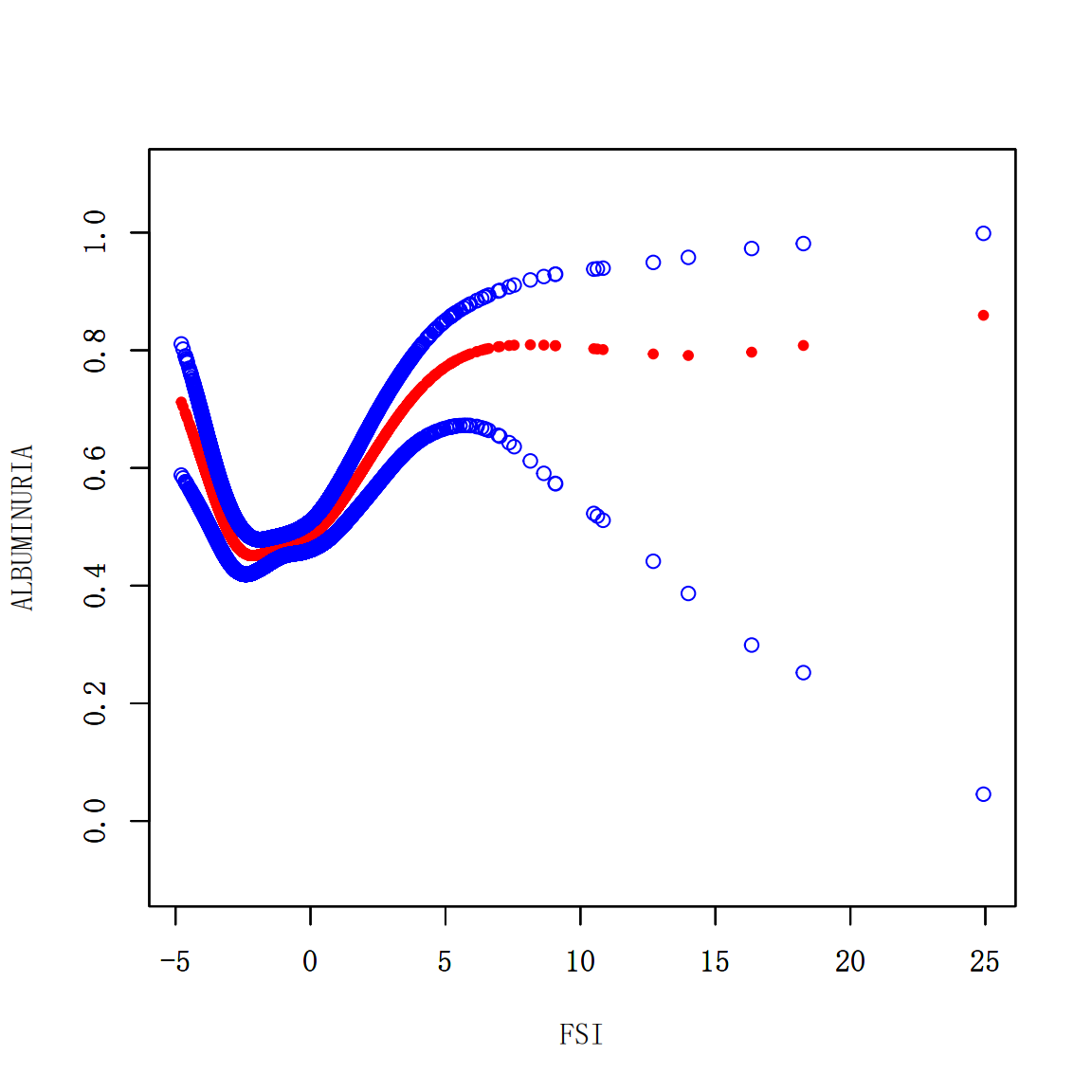

Supplement: S1 Fig — (TIF) [file pone.0337104.s006.tif]

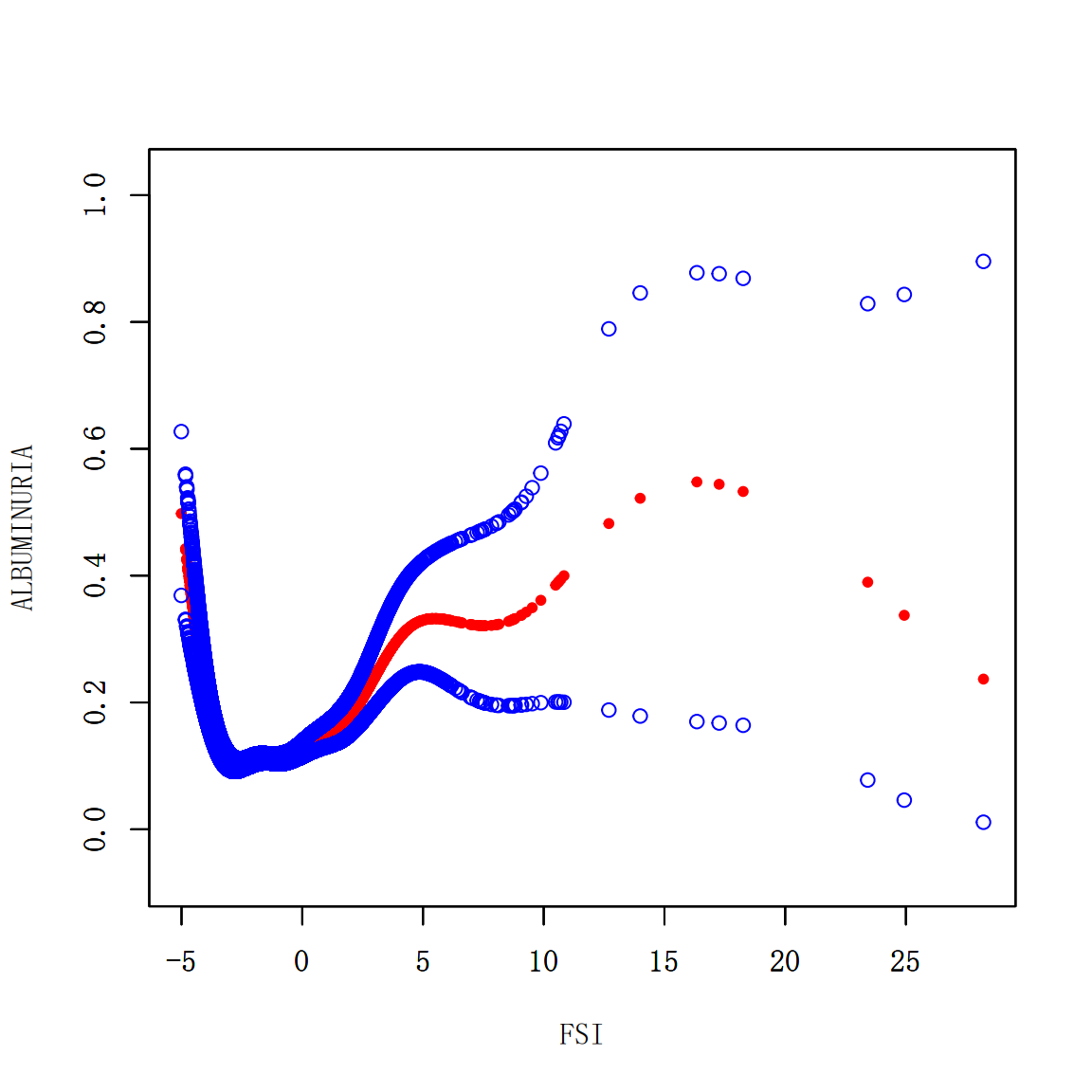

Supplement: S2 Fig — (TIF) [file pone.0337104.s007.tif]

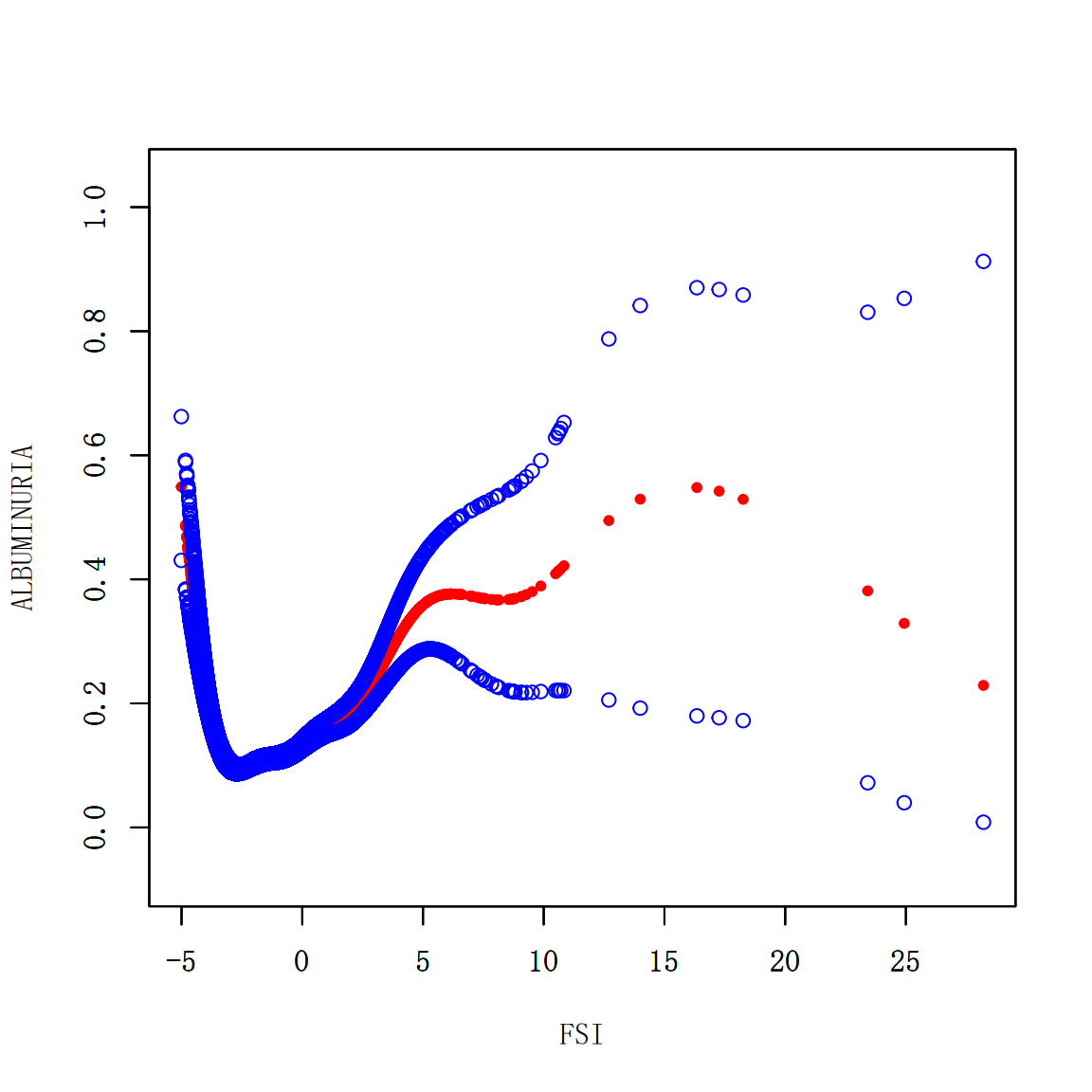

Supplement: S3 Fig — (TIF) [file pone.0337104.s008.tif]
